# Reconstruction of Acquired Labial Adhesions in a Young Adult Female Patient Using Split-Thickness Skin Grafting: A Rare Case

**DOI:** 10.7759/cureus.108175

**Published:** 2026-05-03

**Authors:** Sakshi Bhat, Sandhya Pandey, Mukta Verma, Vijay Kumar

**Affiliations:** 1 Plastic and Reconstructive Surgery, King George's Medical University, Lucknow, IND

**Keywords:** labial adhesions, reproductive age group women, split-thickness skin graft, surgical adhesiolysis, vaginal stenosis

## Abstract

Labial adhesions are rarely encountered in women of reproductive age and are more commonly seen in prepubertal girls or postmenopausal women. They are typically associated with hypoestrogenism, chronic inflammation, trauma, or cicatricial dermatoses. Thus, acquired labial adhesions without identifiable predisposing factors in reproductive-age women are rare and can pose a reconstructive challenge. These adhesions may lead to urinary, gynecological, and psychological morbidity. In this report, we present the case of a 24-year-old woman with progressive narrowing of the vaginal introitus over 5-6 months due to dense labial adhesions. On examination, dense midline fusion of the labia minora with obliteration of the vaginal opening was noted. There was no history of trauma, surgery, sexual activity, or dermatological disease. The patient had a prior history of urinary tract infection, with a positive urine culture reported at another hospital approximately two months before presentation to our center, for which antibiotics were prescribed. A comprehensive evaluation was performed, and hormonal and dermatological causes were excluded. Cystoscopy was performed, which showed a normal urethra and bladder. The patient was catheterized with a 14-French silicone catheter (Sterimed Medical Devices Pvt. Ltd., Haryana, India).

Surgical adhesiolysis was performed, followed by reconstruction of the vaginal introitus using a thin split-thickness skin graft to prevent recurrence and restore normal anatomy. In the postoperative period, an impression of the vaginal cavity was taken using dental compound, and an acrylic mold was fabricated. At the six-month follow-up, there was no evidence of recurrence, and complete healing with satisfactory functional and anatomical outcomes was observed. Early diagnosis and individualized surgical management of rare presentations of labial adhesions in women of reproductive age are important to prevent physical and psychological morbidity.

## Introduction

Labial fusion, also known as labial adhesion, labial synechia, or labial agglutination, refers to the adherence of the labia minora or labia majora along the midline of the vestibule. This can eventually lead to narrowing or obliteration of the vaginal introitus. It may be classified as primary or secondary. Primary labial adhesions are commonly seen in prepubertal girls. Secondary labial adhesions occur most often in postmenopausal women due to estrogen deficiency [1]. Accordingly, this condition is well documented in prepubertal girls, who are in a low-estrogen state, and in postmenopausal women, in whom estrogen deficiency, local inflammation, and poor hygiene are recognized contributing factors.

In contrast, labial adhesions are less frequently seen in women of reproductive age [2], particularly in the absence of prior trauma, female genital mutilation, surgery, radiation therapy, chronic infections, herpes simplex infection, caustic injury, or cicatricial dermatoses such as lichen sclerosus and lichen planus [3].

Clinical presentation ranges from asymptomatic partial fusion to severe adhesions causing urinary retention, recurrent urinary tract infections, menstrual difficulties, dyspareunia, and psychological distress. Patients who do not respond to topical therapy may have dense adhesions requiring manual or surgical separation [3]. We report a rare case of acquired labial adhesions in a young woman with no identifiable predisposing factors, highlighting the role of surgical reconstruction in restoring normal anatomy and preventing recurrence.

Therefore, prompt diagnosis and appropriate management are essential to reduce associated physical and psychological morbidity.

## Case presentation

A 24-year-old woman presented with a complaint of a gradual reduction in the size of the vaginal opening over approximately five months. There was no history of acute pain or bleeding, and no history of vaginal delivery. She reported intermittent per vaginum discharge over the same duration. There was a history of urinary tract infection, with a positive urine culture performed at another hospital approximately two months prior to the presentation at our center. Antibiotics were prescribed, following which the vaginal discharge decreased, and the urinary tract infection resolved about one month before presentation. There was no history of trauma, burns, sexual assault, female genital mutilation, or prior surgical intervention. There was also no history of any known dermatological disorder involving the external genitalia. The patient was not on any long-term medications. Menarche occurred at 13 years of age. Menstrual cycles were regular, with normal flow and no history of dysmenorrhea.

**Figure 1:**
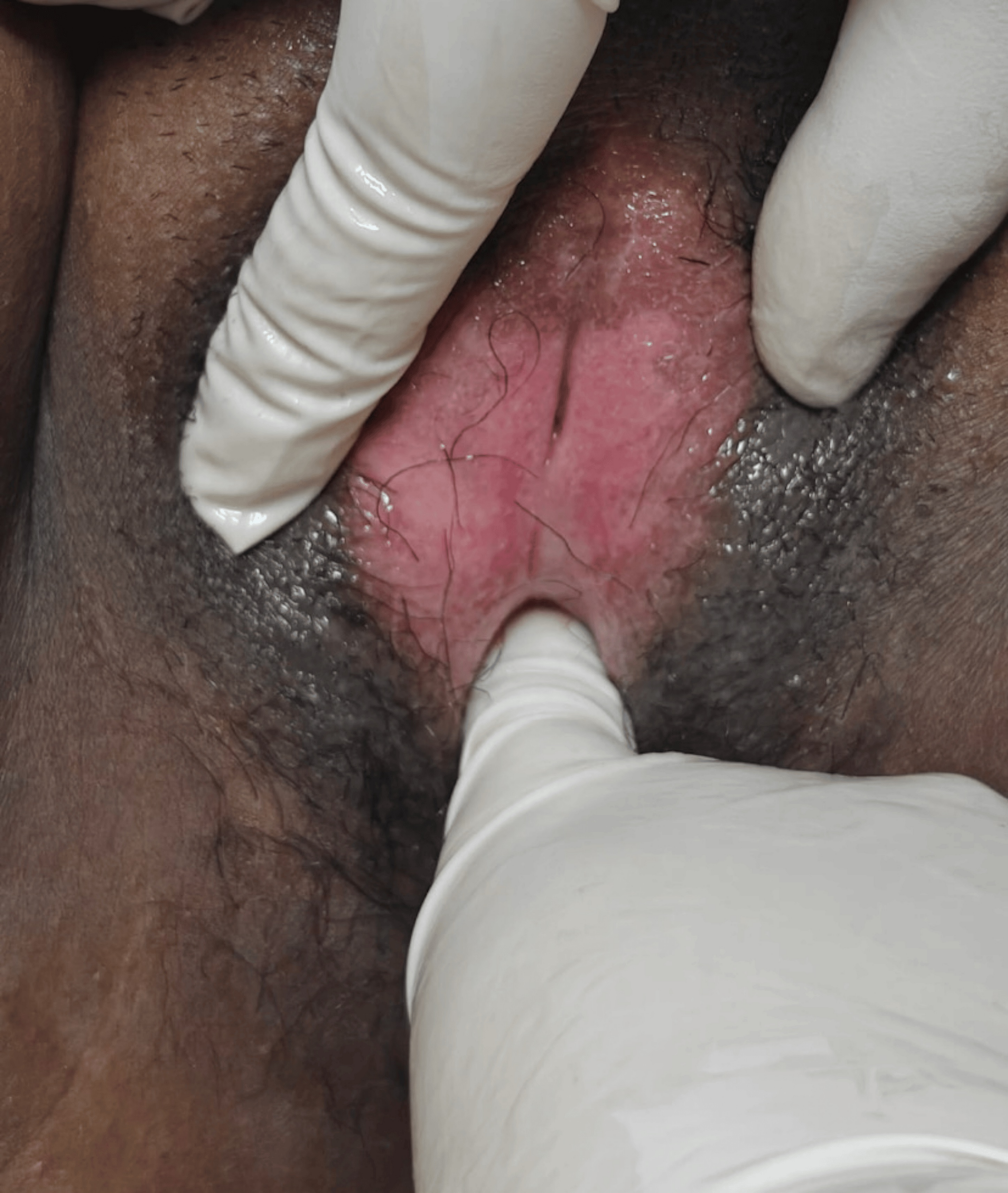
Preoperative image showing dense midline adhesion of the labia minora, obscuring the urinary meatus and vaginal introitus

General and systemic examination revealed no abnormalities. The patient had age-appropriate secondary sexual characteristics. On local examination of the external genitalia, the labia majora appeared normal. The labia minora were densely adherent across the midline, resulting in near-complete obliteration of the vaginal introitus. There were no signs of active inflammation, ulceration, or skin discoloration, as shown in Figure 1.

**Figure 2:**
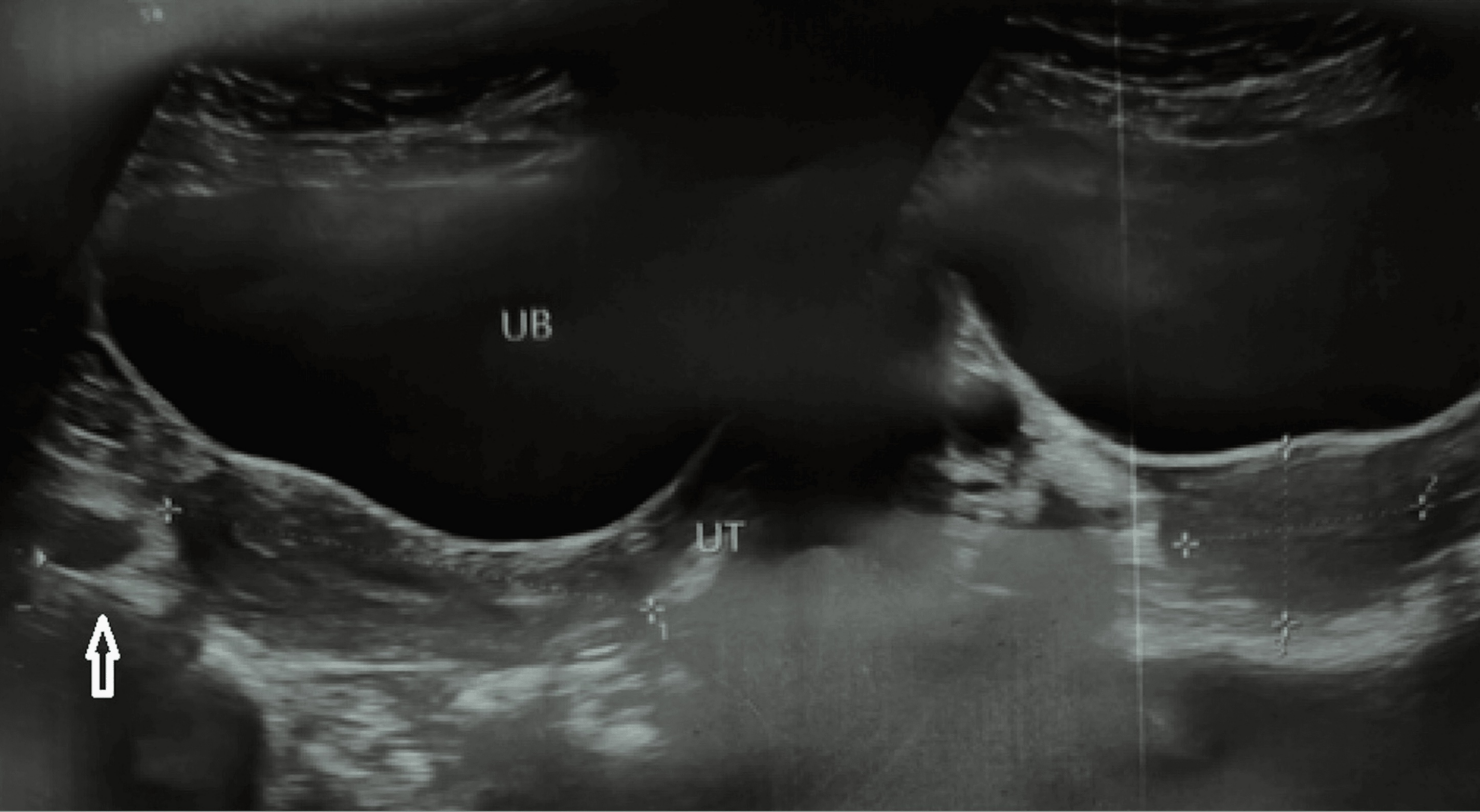
Ultrasonography of the abdomen and pelvis revealing normal uterine and adnexal anatomy, with no evidence of outflow obstruction

Given the unusual presentation, a multidisciplinary evaluation was undertaken. All laboratory investigations, including complete blood count, inflammatory markers, and hormonal profile, were within normal limits. Ultrasonography of the abdomen and pelvis revealed normal uterine and adnexal anatomy, with no evidence of outflow obstruction, as shown in Figure 2. 

Informed consent for surgery was obtained. Given the density of the adhesions and the absence of obvious precipitating factors, a dermatological opinion was sought to exclude underlying cicatricial dermatoses such as lichen sclerosus or lichen planus; both were ruled out.

A urological consultation was obtained to confirm the location and patency of the urethral opening. The urethral meatus was identified, and cystoscopy revealed no abnormalities of the urethra or bladder. The patient was catheterized with a 14-French silicone catheter (Sterimed Medical Devices Pvt. Ltd., Haryana, India).

**Figure 3:**
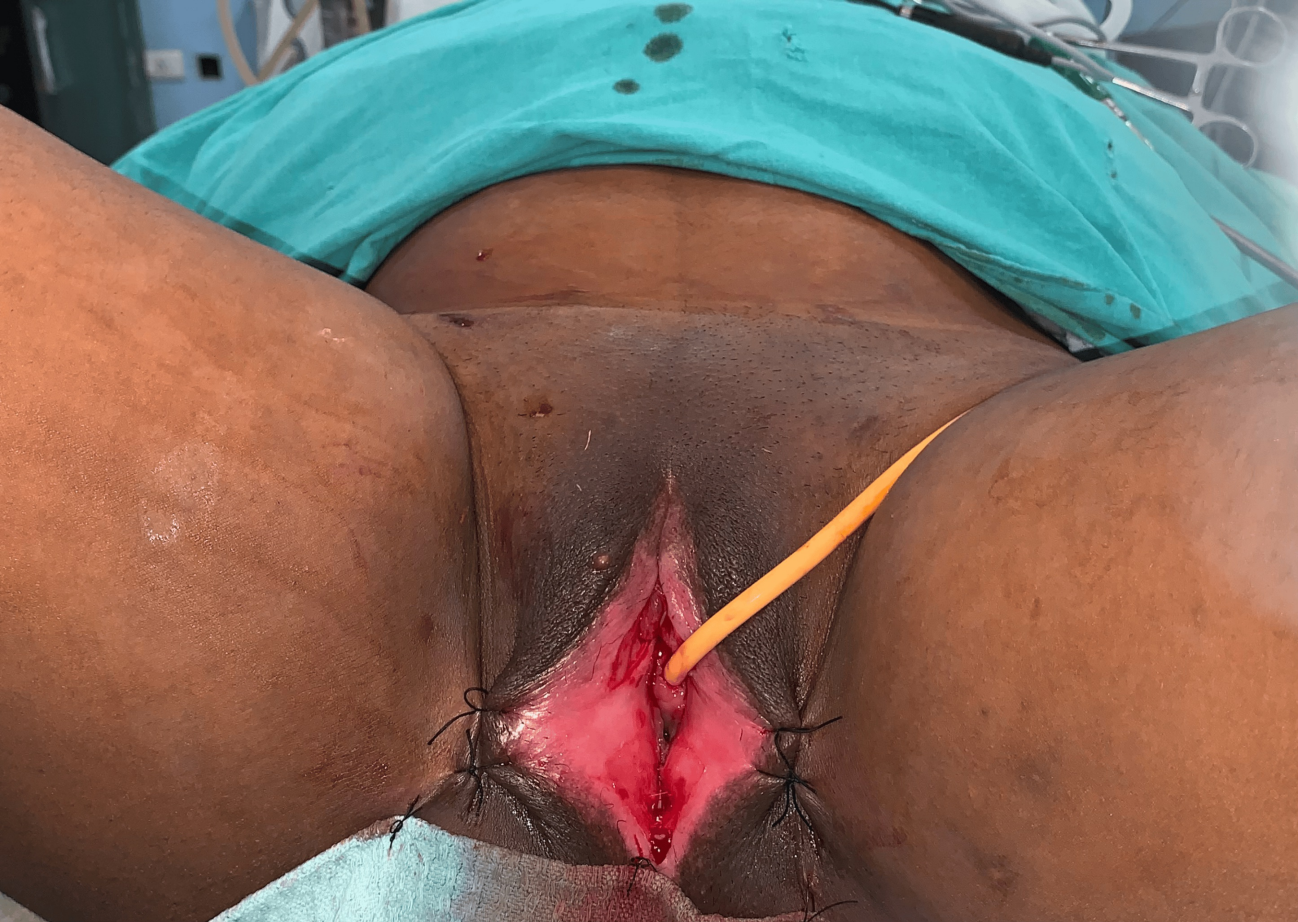
Intraoperative image showing dense adhesions, with release resulting in extensive raw areas over the labia minora

In view of the dense adhesions and associated functional obstruction, surgical intervention was planned. The patient underwent the procedure under spinal anesthesia. Adhesiolysis of the labial adhesions was performed, and the vaginal opening was identified and adequately delineated. The adhesions were dense, and their release resulted in extensive raw areas over the labia minora, as shown in Figure 3. Therefore, healing by secondary intention was considered inappropriate. 

**Figure 4:**
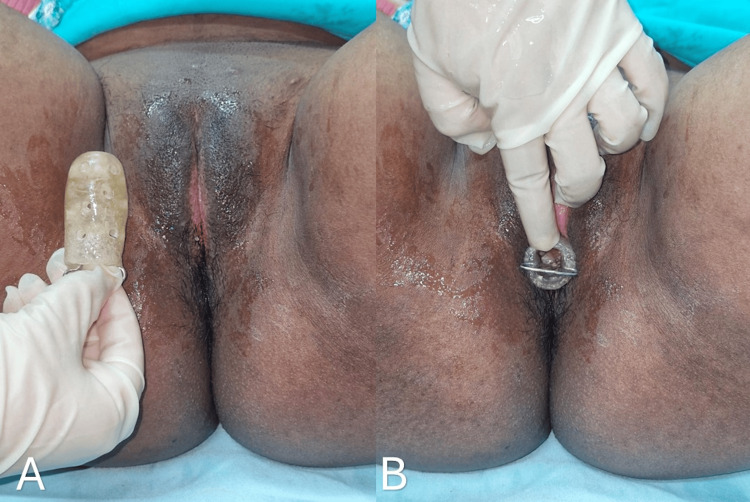
Postoperative image showing the (A) impression of the vaginal cavity taken using dental compound, from which an acrylic mold was fabricated and an (B) acrylic mold placed inside the vaginal cavity

Thus, to prevent recurrence and reconstruct the vaginal introitus, a thin split-thickness skin graft was harvested from the right thigh and secured over the raw area to resurface the defect. Meticulous hemostasis and graft fixation were ensured using 4-0 Vicryl sutures. A Bactigras dressing was applied over the graft. The first dressing was performed on postoperative day 7. An impression of the vaginal cavity was then taken using dental compound, and an acrylic mold was fabricated from the impression, as shown in Figures 4A, 4B.

**Figure 5:**
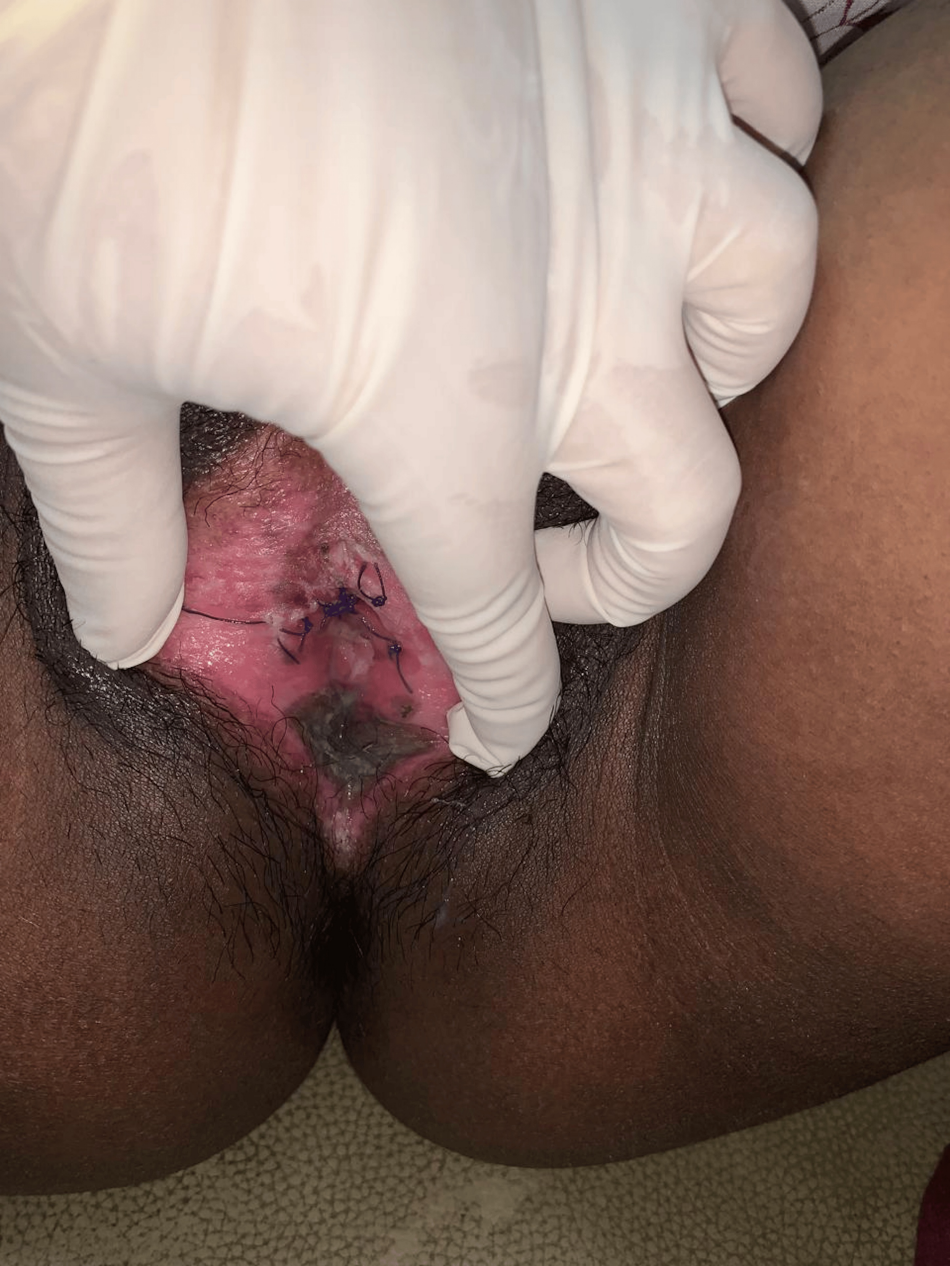
Postoperative image on day 14 showing a well-taken graft

The indwelling catheter was maintained for 14 days before removal. Graft take was satisfactory, as shown in Figure 5.

**Figure 6:**
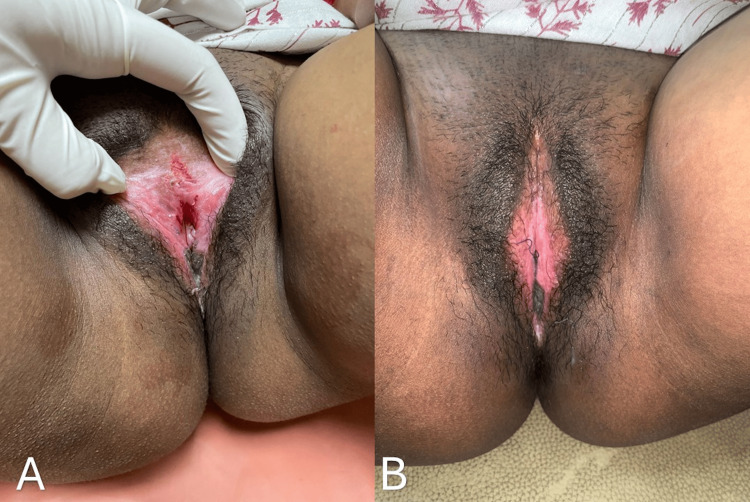
(A, B) Postoperative images at one month showing a well-epithelialized vaginal introitus

The donor site was dressed with Bactigras and healed within 21 days. At the one-month follow-up, the patient had a well-epithelialized vaginal introitus, as shown in Figures 6A, 6B.

**Figure 7:**
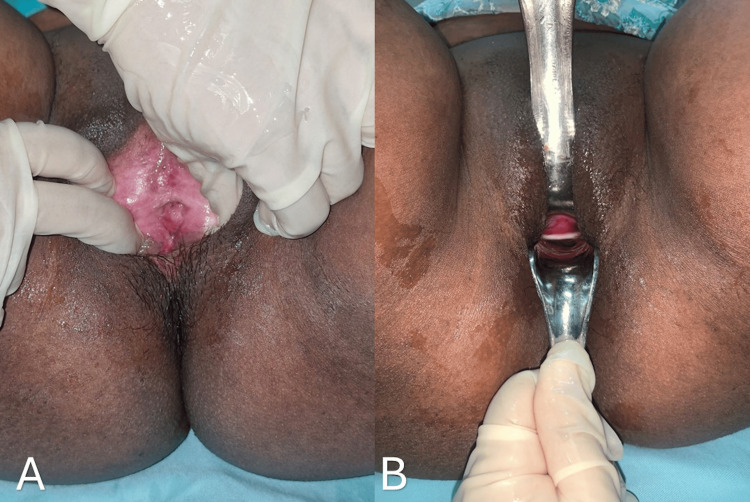
(A, B) Postoperative images at the three-month follow-up showing a well-epithelialized vaginal introitus

At the three-month follow-up, the patient had a well-epithelialized vaginal introitus with no evidence of scarring, stenosis, or recurrence of labial adhesions, as shown in Figures 7A, 7B.

After a satisfactory graft take, the mold is used for six months to one year. Thus, the risk of long-term recurrence is minimal.

## Discussion

Labial adhesions are more frequently encountered in the prepubertal and postmenopausal age groups. Nabi presented a case of a 62-year-old postmenopausal woman with no underlying disease [1]. Labial adhesions in women of reproductive age represent a rare diagnostic and therapeutic challenge. Estrogen deficiency and chronic inflammatory processes are most commonly associated with this condition. Other causes, such as trauma, surgery, infection, or dermatological conditions, are usually observed in young adults. Subclinical inflammation, poor local hygiene, lack of awareness, and delayed presentation may contribute to idiopathic adhesions. Early intervention is important to prevent the progression of idiopathic labial adhesions. 

Singh et al. reported a 32-year-old woman who presented with complaints of an extremely thin urinary stream, intermittent burning micturition, increasing voiding difficulty, and painful menses for the past 2.5 years [2]. Suprapubic cystostomy was performed. Surgical adhesiolysis of the labial adhesions was done, and a small tongue-shaped 2-cm skin flap was raised. Gradual midline dissection was performed to enter the vaginal lumen. A limited left posterolateral episiotomy was performed, and the skin flap was sutured to the margin of the episiotomy incision to reconstruct the vaginal introitus.

Clinical presentation may also vary between individuals. In a study by Mayoglou et al., among 151 patients with labial adhesions, 11 (7.3%) presented with urinary frequency, 30 (19.9%) with urinary tract infections, 13 (8.6%) with vaginitis, and 19 (12.6%) with post-void dribbling [3].

In the present case, the patient had a history of intermittent vaginal discharge and urinary tract infection; however, these occurred after the onset of adhesions, with no prior symptoms before adhesion formation. Subclinical urinary or vaginal infections may have contributed to epithelial denudation, leading to adhesion formation. Despite a comprehensive evaluation, no clear etiological factor was identified.

Management varies depending on the patient’s age, severity of adhesions, and associated symptoms. In prepubertal girls, topical estrogen therapy or gentle manual separation is commonly used. Betamethasone has also recently shown efficacy in separating labial fusion [2]. Bacon noted that patients with labial agglutination may have dense adhesions and often require manual separation when they do not respond to topical therapy [4]. However, in adult patients with dense or complete adhesions, surgical intervention is usually required. Simple adhesiolysis alone is associated with a higher risk of recurrence.

Various reconstructive options described in the literature include local flaps, mucosal advancement, and skin grafting. In this patient, the use of a split-thickness skin graft resulted in successful anatomical outcomes, emphasizing that split-thickness skin grafting reduces the risk of restenosis and recurrence by providing stable epithelial coverage.

Labial adhesions can result in significant physical and psychological morbidity, particularly in young women. Early diagnosis and prompt treatment are essential to prevent urinary, sexual, and psychological complications.

## Conclusions

Acquired labial adhesions in women of reproductive age are rare. They may lead to recurrent perineal infections, acute urinary retention, and dyspareunia. To exclude underlying dermatological, infectious, and hormonal causes, a comprehensive evaluation is essential.

Individualized treatment planning based on clinical presentation is important for appropriate management. Decisions regarding the type of incision and the need for graft or flap reconstruction should be made on a case-by-case basis. Operative intervention in the form of surgical adhesiolysis with reconstruction, such as split-thickness skin grafting, provides effective and long-term results in cases with dense adhesions. Prompt diagnosis and individualized treatment are important to reduce both physical and psychological morbidities associated with this rare condition.
